# Habitat-Adapted Endophytic *Fusarium clavum* EeR24 from the Arava Desert Induces Resistance Against *Fusarium* Wilt of Muskmelons

**DOI:** 10.3390/microorganisms14040871

**Published:** 2026-04-12

**Authors:** Vineet Meshram, Meirav Elazar, Marcel Maymon, Gunjan Sharma, Eduard Belausov, Dana Charuvi, Mahiti Gupta, Soniya Goyal, Surbhi Goel, Stanley Freeman

**Affiliations:** 1 Department of Plant Pathology and Weed Research, The Volcani Institute, Agricultural Research Organization, Rishon LeZion 7528809, Israel; vineetmeshram@anjaneyauniversity.ac.in (V.M.); meirav.elazar@gmail.com (M.E.); marcelma@volcani.agri.gov.il (M.M.); gunjan.sharma@gbu.edu.in (G.S.); 2 Department of Biotechnology, Anjaneya University, Nardaha, Raipur 492101, CG, India; 3 Department of Plant Biotechnology, Gujarat Biotechnology University, GIFT City, Gandhinagar 382355, GJ, India; 4 Institute of Plant Sciences, The Volcani Institute, Agricultural Research Organization, Rishon LeZion 7528809, Israel; eddy@volcani.agri.gov.il (E.B.); charuvi@volcani.agri.gov.il (D.C.); 5 Department of Biosciences and Technology, Maharishi Markandeshwar (deemed to be) University, Mullana-Ambala 133207, HR, India; mahitigupta@gmail.com (M.G.); soni.goyal48@gmail.com (S.G.); 6 Independent Researcher, Charlotte, NC 28202, USA; surbhigoel17@gmail.com

**Keywords:** biocontrol agent, biotic stress, clavicipitaceous endophytes, *F. oxysporum* f. sp. *melonis*, squirting melons

## Abstract

Muskmelon (*Cucumis melo*) is a widely cultivated and economically important fruit crop that is severely affected by *Fusarium* wilt caused by *Fusarium oxysporum* f. sp. *melonis* (race 1.2) (Fom). Conventional management practices have shown limited effectiveness and pose environmental and health risks; therefore, sustainable and eco-friendly alternatives are required to manage this disease. In the present study, 23 endophytic fungal isolates belonging to eight genera were isolated from *Ecballium elaterium* and screened to determine antifungal potential against Fom using an in vitro antagonistic assay. Two endophytic isolates (*Fusarium* sp. EeR4 and *Fusarium clavum* EeR24) exhibited an inhibitory effect against Fom on quarter-strength PDA plates. In growth chamber experiments, *F. clavum* EeR24-colonized melon seedlings and significantly protected plants from wilting compared to non-colonized pathogen-challenged seedlings. Under greenhouse conditions, *F. clavum* EeR24 significantly improved morphological and physiological traits, including plant height, weight, number of leaves, membrane stability, photosynthesis, stomatal conductance, and transpiration, in *Cucumis melo*. Endophytic colonization improved catalase (56%), guaiacol peroxide (47%), and superoxide dismutase activity (25%), and increased flavonoid and phenolic content by 11–59% compared to non-colonized Fom-challenged plants. Lipid peroxidation significantly decreased by 37% and proline accumulation increased by 70% in colonized plants compared to non-colonized plants. Histochemical analysis also indicated that endophytic colonization considerably reduced the levels of H_2_O_2_, O_2_^−^, malondialdehyde, and cell mortality in Fom-challenged plants. In addition, the culture filtrate and organic residues of *F. clavum* EeR24 inhibited the mycelial growth of Fom by 52–58%, respectively. Furthermore, a study on spatial colonization of the endophyte and the pathogen using GFP and RFP tagging indicated that both the endophyte and the pathogen simultaneously colonized the root tissues of *C. melo*; however, the endophyte significantly reduced the pathogenicity of Fom. These results suggest that endophytic *F. clavum* EeR24 may be developed as an effective biocontrol agent for the management of *Fusarium* wilt in melon plants under field conditions.

## 1. Introduction

Muskmelon (*C. melo*) is one of the most popular, widely consumed, and economically important fruit crops, with a total harvested area of 1,068,238 ha and an annual production of approximately 28.5 million tons worldwide [1]. It is ranked fourth in the fresh fruit market and possesses essential vitamins, minerals, nutrients, and several other bioactive compounds that have certain medicinal and nutritional properties [2,3,4]. Muskmelon is extensively cultivated in the Mediterranean region and Southeast Asian countries. In Israel, it is cultivated in the deserts of the Jordan and Arava valleys, where extreme climatic conditions prevail, which are major limiting factors to plant growth and development [5]. Muskmelon is susceptible to soilborne diseases caused by a variety of pathogenic microorganisms, which may result in 100% yield losses [6,7]. *Fusarium* wilt of melon caused by Fom is a prevalent severe fungal disease that significantly impacts the yield and commercial value of muskmelon. Fom is a soilborne pathogen that invades the vascular system of melons, eventually resulting in seedling and plant mortality [8]. Fom can survive persistently in the soil for several years by producing conidia and chlamydospores, which then serve as the primary source of inoculum in the following year. Thus, the disease is particularly severe in monocultured melon fields and remains a major problem in melon cropping systems [9].

Traditional control strategies, including the use of fungicides, crop rotation, and development of wilt-resistant cultivars, have been suggested to control melon *Fusarium* wilt; however, these strategies have provided limited protection against *Fusarium* wilt, along with hazardous effects on the environment and human health [10]. The use of resistant cultivars has also led to the development of new virulent races in specific locations. In Fom, four common races (0, 1, 2, and 1.2) exist worldwide, and to date no known resistant field cultivars are available to this pathogen [11,12,13]. Therefore, alternative avenues need to be explored to develop robust, sustainable, and environmentally friendly strategies to manage and control *Fusarium* wilt in muskmelons [12]. Grafting of melon seedlings onto cucurbit rootstock is one of the most effective approaches for controlling *Fusarium* wilt, and is widely practiced in countries like Israel, Spain, and South Korea. However, due to the high price of grafted transplants, other plausible approaches need to be investigated [14]. Biological control offers an attractive alternative method for protection of melon against *Fusarium* wilt. A nonpathogenic mutant strain of Fom significantly colonized the seedling roots and stem tissues and reduced the mortality of melon seedling in cross-protection experiments [15]. A bioorganic fertilizer made from organic fertilizer and antagonistic microorganisms decreased the incidence of *Fusarium* wilt and increased melon yield [16]. Similarly, *Trichoderma polysporum* in combination with liquid compost significantly decreased *Fusarium* wilt of melon and increased fruit production by 100% [10]. A talc-based formulation of two strains of *Pseudomonas putida* effectively suppressed the growth of *Fusarium* wilt of muskmelon [17]. Several antagonistic strains, such as *Cadophora*, *Chaetomium*, *Fusarium*, *Meyerozima*, *Penicillium*, and *Trichoderma* strains, have been proven as effective biocontrol agents against *Fusarium* wilt in controlled laboratory or greenhouse conditions [6,10,18,19,20]. Hence, using beneficial microorganisms to manage plant disease not only enhances agricultural yield and product quality, but also reduces reliance on chemical pesticides [18].

Endophytes are a ubiquitous, polyphyletic group of microorganisms that spend an entire or significant part of their life cycle within living tissues of host plants without causing detrimental impacts [21,22,23]. Endophytes are well known to provide fitness benefits to their host plants by mediating abiotic and biotic stress tolerance, inducing resistance [6,10,18,24], regulating nutrition acquisition [20], decreasing water consumption [25,26], improving photosynthetic machinery [27], inducing antagonism, antibiosis, and mycoparasitism [19,28,29,30,31], preventing ROS production [5,32], producing antioxidant enzymes, [5,33] and expressing genes involved in homeostasis [34]. Based on their evolutionary history, taxonomy, host plant specificity, and ecological roles, fungal endophytes have been categorized into two principal groups: clavicipitaceous and non-clavicipitaceous endophytes [35,36]. Clavicipitaceous endophytes, which are restricted to certain grass species, belong to class 1 and consist of a limited number of closely related species. These fungi exhibit vertical transmission, passing directly from one generation to another. In contrast, non-clavicipitaceous endophytes, which are found within a broader range of host plants, including bryophytes, ferns, gymnosperms, and angiosperms, are further divided into class 2, class 3, and class 4. These endophytes exhibit both horizontal and vertical modes of transmission [37]. Interestingly, certain studies have suggested that class 2 fungal endophytes adapt native plants to various abiotic and biotic stresses via habitat-specific fungal adaptation, i.e., fungal species isolated from plants growing in a particular area with high levels of stress are specially adapted to improving host stress tolerance [5,7,27,35]. For example, *Dichanthelium lanuginosum* (panic grass) thrives in geothermal soils, is symbiotic with the fungus *Curvularia protuberata*, and confers thermotolerance to this grass at elevated temperatures [38]. It was also shown that the *C. protuberata* endophyte can asymptomatically colonize genetically distant plants (tomato, rice, wheat, squash) and confer heat tolerance equivalent to that observed in panic grass [39]. Fungal endophytes such as *Alternaria* and *Trichoderma* isolated from naturally growing plants in salinized soil in Saskatchewan, Canada, conferred salt and drought tolerance to tomato [27]. Similarly, endophytic *Acremonium sclerotigenum* and *Sarocladium implicatum* isolated from a wild wheat variety (Sharon goat grass) protected wheat plants from drought by altering the physiological responses of the host plant to water stress [25]. Endophytic *Trichoderma phayaoense* isolated from *Chromolaena odorata* (Siam weed) growing in the forest of Phayao province, Thailand, inhibited the mycelial growth of *Stagonosporopsis cucurbitacearum* and *Fusarium equiseti*, which cause gummy stem blight and wilt of muskmelon, respectively. In addition, *T. phayaoense* also tolerated a fungicide (metalaxyl) at recommended dosages for field applications, and thus can be used together with the fungicide to manage gummy stem blight and wilt of muskmelon [7].

The endophytic fungal isolate *F. clavum* EeR24, isolated from *Ecballium elaterium* growing in the Arava Valley, Israel in a previous study, mitigated salt stress in muskmelons by improving morphological and physiological traits, decreased absorption of Na^+^ and Cl^−^ ions, and enhanced production of antioxidant enzymes, osmoprotectants, and growth hormones [5]. Therefore, isolation, preservation, and characterization of these fungal endophytes surviving within plants under stressed environments are of paramount biotechnological and agricultural importance [26,40]. In this study, we comprehensively assessed the ability of fungal endophytes isolated from *E. elaterium* to inhibit the growth of Fom responsible for causing *Fusarium* wilt in muskmelon. After preliminary antagonistic and growth chamber assays, the endophytic isolate *F. clavum* EeR24 was selected for greenhouse experiments. Although there are previous reports that investigated the biocontrol potential of endophytes against Fom [6,10], to the best of our knowledge, this is the first study on exploiting the potential of habitat-adapted fungal endophytes of *E. elaterium* isolated from the Arava valley in Israel as a biocontrol agent against Fom in melon plants.

## 2. Materials and Methods

### 2.1. Plant Sample Collection and Isolation of Fungal Endophytes

Healthy and mature root samples of *E. elaterium* (aka squirting melons) growing in the Arava Valley, Israel were collected in August 2017. Isolation was performed within 24 h of sample collection. The samples were washed thoroughly under running tap water for 15 min to remove adhered debris. Thereafter, the samples were sequentially surface-sterilized with 70% ethanol for 10 s followed by 1% sodium hypochlorite for 2.3 min and then rinsed thrice with sterile distilled water (SDW). The surface-sterilized samples were then cut into 2–5 mm segments with the help of a sterile blade and placed on potato dextrose agar (DifcoTM, Becton, Dickinson and Company, Sparks, MD, USA) plates supplemented with chloramphenicol (PDAC) (250 mg/L) (Arcos Organics, Saddle Brook, NJ, USA). The plates were then incubated at 26 ± 2 °C for 7–10 days, with 12 h light and dark cycles. Individual colonies were picked from the edge of an advancing colony and were further purified by single spore isolation. The pure endophytic isolates obtained were stored in glycerol stocks at −80 °C until further examination and characterization [21].

### 2.2. Fungal DNA Extraction

Genomic DNA was extracted from all collected fungal isolates (Table 1) as per Meshram et al. (2023) [5]. The extracted DNA was visualized under UV light (Enduro GDS, Labnet International, Edison, NJ, USA) after separation in 1.2% agarose gels (SeaKem LE Agarose, Waltham, ME, USA) stained with ethidium bromide. The purity and quantity of DNA were determined using an ND-1000 spectrophotometer (Thermo Fisher Scientific, Waltham, MA, USA) at 260 and 280 nm [5].

### 2.3. Arbitrarily Primed PCR (ap-PCR) Amplification of Fungal DNA

Ap-PCR was performed on DNA of all 23 fungal isolates with three of the repeat-motif primers—(CAG)_5_, (GACA)_4_ and (GACAC)_3_ (Integrated DNA Technologies, Coralville, IA, USA)—as per [5]. The amplification products were separated in 1.8% agarose gels (SeaKem LE Agarose, Lonza Bioscience, Basel, Switzerland) in 1X Tris-acetate–EDTA buffer and run at a constant voltage of 80 V for 1.5 h. Representative isolates were chosen from those that had identical banding patterns after Ap-PCR amplification. Ap-PCR was repeated twice for reference isolate DNA to verify reproducibility of the results [5,41].

### 2.4. ITS-Based Identification of Fungal Endophytes

For molecular identification of fungal endophytes, the universal internal transcribed spacer (ITS) primers ITS1 and ITS4 were used to amplify fungal ITS regions (ITS1-5.8S- ITS2). The amplified products (~500 bp) were resolved on 1.2% agarose gels and imaging was performed under UV light (Enduro^TM^ GDS, Labnet International Inc, Edison, NJ, USA) using a documentation system (Enduro GDS analysis software). The products were purified using a NucleoSpin gel and PCR cleanup kit (Macherey-Nagel GmbHand Co., Backnang, Germany) and the amplified products were sequenced at Macrogen Inc (Amsterdam, The Netherlands). Further, the obtained sequences were homology-searched using NCBI BLAST 2.17.0 (http://blast.ncbi.nlm.nih.gov; default parameters). Identification of the fungal endophytes (to the genus level) was ascertained based on maximum query coverage and score in the BLAST results and further verified using the UNITE database (https://unite.ut.ee/analysis.php accessed on 3 February 2026) [5].

### 2.5. Screening of Fungal Endophytes for Antifungal Activity

Fungal endophytes were screened for their antagonistic activity against Fom by a modified dual-culture method [42]. Petri dishes containing quarter-strength potato dextrose agar (PDA) were inoculated with actively growing mycelial plugs (5 mm) of endophytic fungi and Fom 4 cm apart from each other. Petri dishes inoculated with Fom and an agar plug on opposite sides served as controls. The plates were sealed with double-layered parafilm and incubated in the dark at 28 ± 1 °C for 7 days, and treatments were replicated in triplicate. Antagonistic effects were determined by calculating the percentage of radial growth inhibition (PGRI) using the formula: (R_1_−R_2_)/R_1_ × 100, where R_1_ is the radial growth of Fom without the fungal endophyte and R_2_ is the radial growth of Fom co-inoculated with the fungal endophyte. An analysis of variance (ANOVA) of the PGRI values was carried out seven days post incubation to determine differences between treatments based on Tukey’s significance test (*p* < 0.05). Furthermore, the type of antagonism exhibited by the fungal endophytes towards Fom was categorized into three categories i.e., competition for substrate (Type A), antibiosis (Type B) and mycoparasitism (Type C), with A = deadlock with mycelial contact, B = deadlock at a distance, and C = overgrowth without initial deadlock, as described by Huang et al. 2020 [43]. Fungal endophytes exhibiting antibiosis or Type B interaction in in vitro assays were selected for further studies.

### 2.6. Plant Cultivar and Treatments with Endophytic Fungi Fusarium clavum EeR24 and Fusarium sp. EeR4

The Ra’anan muskmelon variety (susceptible to wilting by Fom) was used to study the effect of endophyte colonization on seedlings under biotic stress using four-week-old seedlings, kindly provided by Hishtil Nehalim Nursery, Nehalim, Israel. Endophytic *F. clavum* EeR24 and *Fusarium* sp. EeR4 exhibiting Type B interaction with Fom were selected for further studies. Conidia of selected fungal endophytes and Fom were separately produced in *Fusarium* minimal medium (FMM), as previously described by [5]. Conidial concentration was adjusted to 1 × 10^6^ conidia per milliliter. The following treatments (Treatment 1–3) were individually set up by soaking the melon seedling roots in a conidial suspension (1 × 10^6^ per mL) in saline solution and shaken gently on an orbital shaker at 150 rpm for 1 h, then incubated under growth chamber or greenhouse conditions.

Treatment 1: Non-colonized seedlings: control/reference.

Treatment 2: Endophyte-colonized seedlings.

Treatment 3: Non-colonized seedlings challenged with pathogen: Fom.

Treatment 4: Endophyte-colonized seedlings challenged with pathogen.

For treatment 4, melon seedlings were first colonized with fungal endophytes, incubated for 7 days at 25 ± 1 °C, and then treated with a conidial suspension (1 × 10^6^ conidia/mL) of Fom [44].

### 2.7. Effect of Selected Fungal Endophytes (Fusarium clavum EeR24 and Fusarium sp. EeR4) on Melon Seedlings in Growth Chamber Assays

The ability of selected fungal endophytes in conferring Fom disease tolerance to melon seedlings was assessed in a controlled environment (growth chamber) using a randomized block design experiment. Endophyte-colonized and non-colonized melon seedlings (2 weeks old) from different treatments (as mentioned in Section 2.6) were planted in sterile double-decker magenta boxes (14 × 7 × 7 cm) containing equivalent amounts (35 g) of sterile vermiculite (Agrekal, Hof HaCarmel, Israel) in the upper chamber and Hoagland solution in the lower one. Plants were grown under a 12 h light regime at 24 ± 2 °C, 60% relative humidity (RH), and photosynthetic photon flux density of 300–400 μmol/m^2^/s for the next three weeks. After three weeks of exposure, plants were harvested and growth parameters (plant height, plant fresh weight), infection rate, and control efficacy were determined [43,45]. Each experiment contained three replicates per treatment, and experiments were conducted three times.

### 2.8. Effects of Fusarium clavum EeR24 on Melon Seedlings Under Greenhouse Conditions

Seven days after colonization, endophyte-colonized (*F. clavum* EeR24) and non-colonized melon seedlings were challenged with Fom, as described earlier in Section 2.6. The development of Fom-challenged and water control plants grown in the absence or presence of the endophytic fungus was monitored over the next three weeks.

#### 2.8.1. Morphological Parameters

Three weeks after exposure to Fom under greenhouse conditions, the length and fresh weight (FW) of roots, shoots, leaves, and whole plants were measured. Dry weight (DW) was measured after drying the samples in a hot-air oven at 70 °C for 48 h [26,33]. The fresh leaves of each plant were immediately frozen in liquid nitrogen and stored at −80 °C for further biochemical analyses. Roots, stems, and leaves were collected from six different plants per treatment to measure morphological growth parameters. The plants were also monitored regularly for the appearance of wilting symptoms. Disease severity was scored using a 0–9-point scale at 21 dpi [44]. Percentage disease incidence (PDI) and percentage disease severity (PDS) for melon seedlings 21 days postinoculation with *Fusarium oxysporum* f. sp. *melonis* were calculated using the following formulas:

PDI (%) = x/N × 100

PDS (%) = Σ(a + b)/N.Z × 100

where Σ(a + b) = sum of infected seedlings and their corresponding score scale, N (total number of sampled seedlings (6), Z (highest score scale (9), and x (number of infected seedlings (6).

#### 2.8.2. Physiological Parameters

Membrane stability index (MSI), relative water content (RWC), lipid peroxidation, proline levels, and gas exchange parameters were measured. MSI was determined by measuring the electrical conductivity of leaves, as described by [25]. RWC was measured by weighing the FW, DW, and turgid weight (TW) of leaves, as described by [5]. Lipid peroxidation was determined by measuring malondialdehyde (MDA) formation using the thiobarbituric acid method, as described by [46]. Free proline content was measured according to the method described by [47].

To analyze gas exchange parameters, net photosynthesis (P_N_), stomatal conductance (Gs), and transpiration rate (Tr) were quantified in fully developed leaves of melon seedlings under normal and stressed conditions using an LI-6800 portable photosynthesis system (LI-COR Inc., Lincoln, NE, USA), following the protocol of [48]. Three weeks later, six leaves from each treatment were sampled for evaluation. A steady state of reference carbon dioxide (400 ppm) was maintained before taking the measurements. Photosynthetic parameters were measured at 1200 μmol photons m^−2^ s^−1^ (90% red, 10% blue) and a CO_2_ concentration of 400 μmol/mol at 24 °C, and expressed per unit leaf area.

#### 2.8.3. Biochemical Parameters

Antioxidant enzyme and non-enzyme activities along with pigment production were determined. For enzymatic activity, leaves (0.5 g) from all treatments were crushed into fine powder and homogenized in a pre-chilled pestle–mortar in 5 mL of extraction buffer containing 0.1 M phosphate buffer (pH 7). The homogenate was centrifuged at 10,000× *g* for 10 min at 4 °C. The supernatant was collected and used to determine catalase (CAT; EC 1.11.1.6), guaiacol peroxidase (GPX; EC 1.11.17), and superoxide dismutase (SOD) activities. CAT activity was estimated according to [34] by measuring the decomposition of H_2_O_2_ at 240 nm. GPX activity was determined according to [49] based on tetrahydro-guaiacol formation at 470 nm. SOD activity was determined by using the photoinhibition of nitro-blue tetrazolium (NBT) assay at 560 nm absorbance according to [50]. Protein content of the enzyme mixture was measured as previously described [51].

For extraction and estimation of total pigments and phenolic and flavonoid content, approximately 0.1 g of leaves was finely ground and extracted in the dark with 10 mL of ethanol and then centrifuged at 8000× *g* for 10 min. The concentration of chlorophyll a, chlorophyll b, total chlorophyll, and carotenoids were determined according to the method described by [52], by measuring the absorbance at 663, 653, and 470 nm using a Tecan Spark™ 10M multimode microplate reader (Spectro V-12, MRC Lab, Holon, Israel). Total phenolic content was quantified using the Folin–Ciocâlteu procedure previously described by [40]. The absorbance was recorded at 765 nm and expressed in milligrams gallic acid (GAE)/g. Furthermore, total flavonoids were quantified according to [53]. The OD was recorded at 415 nm. Quercetin was used as a standard, and values are expressed as mg QR/g.

### 2.9. In Situ Detection of H_2_O_2_, Lipid Peroxidation, Superoxide Accumulation, and Cell Mortality

Histochemical detection of hydrogen peroxide production was detected by the 3,30-diaminobenzidine (DAB) staining method. Formation of brown spots on the leaves indicated H_2_O_2_ production due to DAB polymerization. Estimation of aldehydes produced by peroxidation of membrane lipids was performed using Schiff’s reagent. Formation of pink spots indicated lipid peroxidation. Cell death was determined using Evans blue dye, where necrotic lesions after staining dye developed as blue spots on leaves. Similarly, superoxide (O_2_^−^) accumulation was localized by formation of blue spots suggesting formazan deposits after infiltrating the leaves with NBT [5,54].

### 2.10. Fungal Transformation, Green and Red Fluorescence Protein Labeling, and Localization of Endophytic F. clavum EeR24 and Fom in Melon Seedlings

Fungal transformations were performed using GFP plasmid pSK1019 [55] and RFP plasmid pCA56 [56]. Fom was transformed using GFP plasmid pSK1019 following *Agrobacterium*-mediated transformation, whereas transformation of endophytic *F. clavum* EeR24 with RFP plasmid pCA56 was previously described [5]. To localize the colonization pattern, conidia of both GFP-labeled and RFP-labeled fungal isolates were individually cultured for 7 days on 50 mL FMM supplemented with hygromycin B (100 μg/mL). Conidia were then harvested and different treatments conducted as previously described in Section 2.6. Melon seedlings were planted in magenta boxes and incubated in a growth chamber at 24 ± 1 °C for three weeks. Colonization patterns in melon seedlings were evaluated at 3, 7, 14, and 21 days after inoculation using an Olympus IX 81 (Tokyo, Japan) inverted laser scanning confocal microscope (FLUOVIEW 500) equipped with a 561 nm laser. RFP and GFP were excited and then emission was collected through a BA 570–620 nm filter. The images were color-coded red for RFP and green for GFP. The transmitted light images were obtained using Nomarski differential interference contrast.

### 2.11. Culture Filtrate Production, Partial Purification, and GC–MS Analysis of Bioactive Compound Produced by F. clavum EeR24

Liquid culture of the endophytic fungus was prepared on potato dextrose broth following the procedure of [21]. Thereafter, a liquid–liquid extraction procedure was adopted for extracting bioactive metabolites from culture filtrate of the endophytic fungus. The cell-free culture filtrate was sequentially extracted with ethyl acetate, chloroform, and hexane. The obtained organic layers were pooled and then evaporated using nitrogen blowout to obtain ethyl acetate, chloroform, and hexane extract residues. Stock solutions (1 mg/mL) of all three residues were prepared in methanol.

To evaluate the toxicity of different organic residues towards Fom, a food poison assay was conducted. The stock solutions of culture filtrate and organic residues were added in appropriate concentration to molten PDA medium in order to achieve a final concentration of 0–10 µg/20 mL of PDA, which was then aseptically poured into 90 mm petri dishes and allowed to solidify. The control plates were dispensed only with methanol and PDB. Plates were then inoculated with actively growing mycelial discs of Fom. Plates were incubated at 26 ± 2 °C for one week. The mycelial growth of Fom in test and control plates based on diameter and inhibition was calculated, as described previously in Section 2.5. Furthermore, the biocontrol efficacy of the bioactive organic residue (ethyl acetate) of endophytic *F. clavum* EeR24 was evaluated against Fom under in vivo conditions in magenta boxes following the procedure described in Section 2.7. For this purpose, 1 mL of organic residue (1 mg/mL) was added for every 100 mL of water. Growth parameters and infection rate were measured three weeks post exposure to Fom.

Based on the food poison assay, GC–MS analyses of the bioactive residue, i.e., ethyl acetate residue, was carried out using Agilent Technologies 7890B GC system coupled with Agilent Technologies 5977A MSD software (Wilmington, DE, USA) with column HP-5MS (30 m × 250 µm × 0.25 µm). The instrument was set to an initial temperature of 40 °C for 3 min. The oven temperature was then raised to 300 °C at an increment rate of 10 °C/min and maintained for 1 h. Injection port temperature was set to 220 °C. The samples were injected in splitless mode at 100 mL/min. The obtained compounds were then identified based on high-quality matching (above 60% similarity) using the database of National Institute of Standards and Technology for compounds (NIST14) [57].

## 3. Results

### 3.1. Isolation and Identification of Fungal Endophytes

A total of 23 endophytic fungal isolates representing eight fungal taxa were isolated from root tissues of *E. elaterium*. All the isolates sporulated and were identified as different taxa based on their morphological characteristics. Most of the fungal isolates belonged to Ascomycota (86.95%), followed by Deutromycetes (8.69%) and Zygomycetes (4.34%). Among the taxa identified, *Fusarium* spp. (43%) were the most predominant colonizers followed by *Alternaria* (17.4%), *Aspergillus* (13.04%), and *Cladosporium* spp. (8.69%), whereas *Curvularia*, *Mortierellales, Plectosphaerella*, and *Phoma* were the least frequently isolated fungal taxa isolated within this study (Table 1).

### 3.2. Genetic Diversity of the Fungal Isolates

Amplification products were obtained for all 23 endophytic isolates collected in this study using three ap-PCR primers: (CAG)_5_, (GACA)_4_, and (GACAC)_3_. A high level of genetic diversity was observed, categorizing the isolates into 19 distinct groups. The representative isolates were then selected from the genetically distinct groups for further identification based on ITS gene sequencing (Table 1, Supplementary Figure S1).

### 3.3. Screening of Fungal Endophytes for Antifungal Activity

**Figure 1:**
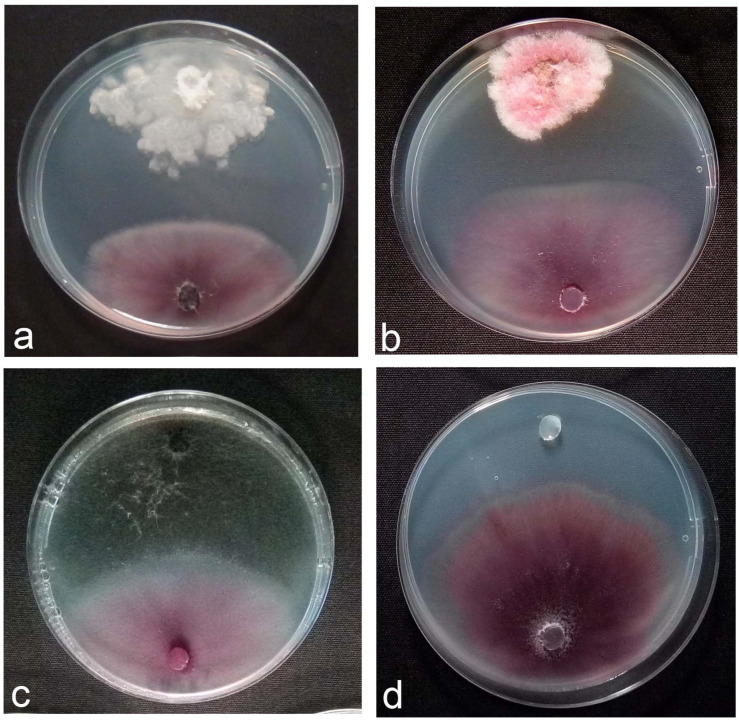
In vitro antagonistic activity of fungal endophytes towards *Fusarium oxysporum* f. sp. *melonis* (race 1.2) (Fom). (**a**,**b**): endophytic isolates *F. clavum* EeR24 and *Fusarium* sp. EeR4 exhibiting antibiosis (Type B interaction), (**c**): endophyte exhibiting mycoparasitism (Type C interaction) towards *Fusarium oxysporum* f. sp. *melonis* (race 1.2), (**d**) control plate with *Fusarium oxysporum* f. sp. *melonis* (race 1.2) growing without any antagonistic fungus.

Varying degrees of mycelial growth inhibition of Fom were observed with the representative antagonistic fungal endophytes. Endophytic *Fusarium* sp. EeR5 and *Alternaria* sp. EeR28 had the maximum inhibitory effect on mycelial growth of Fom, with a reduction of 34%, followed by *Fusarium clavum* EeR24, *Fusarium* sp. EeR10, and *Fusarium* sp. EeR17, with an inhibition of 31% compared to control (Figure 1). Endophytic isolates of *Alternaria* sp. EeR25, *Curvularia* sp. EeR20, *Phoma* sp. EeR22, *Aspergillus* sp. EeR27 and *Fusarium* sp. EeR1 expressed significantly lower mycelial growth inhibition, with reductions in the range of 21–29% (Table 1). The interactions between fungal endophytes and Fom in dual cultures on PDAC plates is also shown in Figure 1. Three classes (A, B, and C) of interactions were observed. Endophytic isolates *Fusarium clavum* EeR24 and *Fusarium* sp. EeR4 exhibiting class B interaction (deadlock at a distance) with the pathogen were considered active and selected for in vivo growth chamber assays against Fom in planta.

### 3.4. Effect of Selected Fungal Endophytes on Melon Seedlings in Growth Chamber Assays

Statistically significant differences in growth parameters were observed between *F. clavum* EeR24-colonized and non-colonized plants in the presence and absence of Fom infection. However, no significant differences were observed in the case of *Fusarium* sp. EeR4-colonized plants with Fom infection. *F. clavum* EeR24-colonized plants challenged with Fom exhibited significantly higher plant biomass and height compared to that of non-colonized plants (Table 2). After three weeks of Fom infection, endophytic colonization with *F. clavum* EeR24 increased fresh plant biomass by 91% and height of melon seedlings by 32% in comparison to non-colonized Fom-infected plants. Furthermore, the control efficacy of *F. clavum* EeR24 against Fom infection was 77% compared to 2% for *Fusarium* sp. EeR4 (Table 2). Thus, endophytic fungal isolate *F. clavum* EeR24 was selected for further greenhouse experiments and characterization.

### 3.5. Effect of F. clavum EeR24 on Melon Seedlings Under Greenhouse Conditions

#### 3.5.1. Morphological Parameters

**Figure 2:**
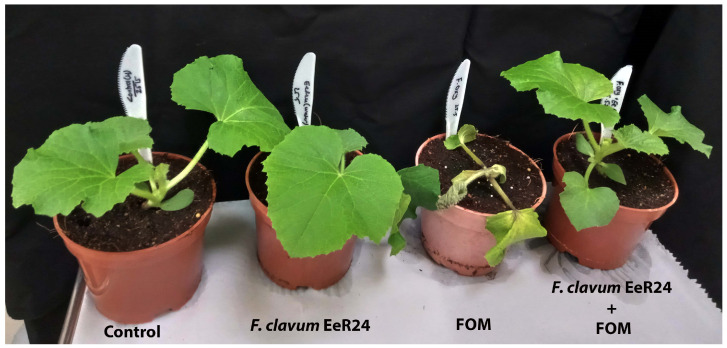
Effect of fungal endophyte *Fusarium clavum* EeR24-colonization on melon plants infected with or without *Fusarium oxysporum* f. sp. *melonis* (race 1.2) under greenhouse conditions.

**Figure 3:**
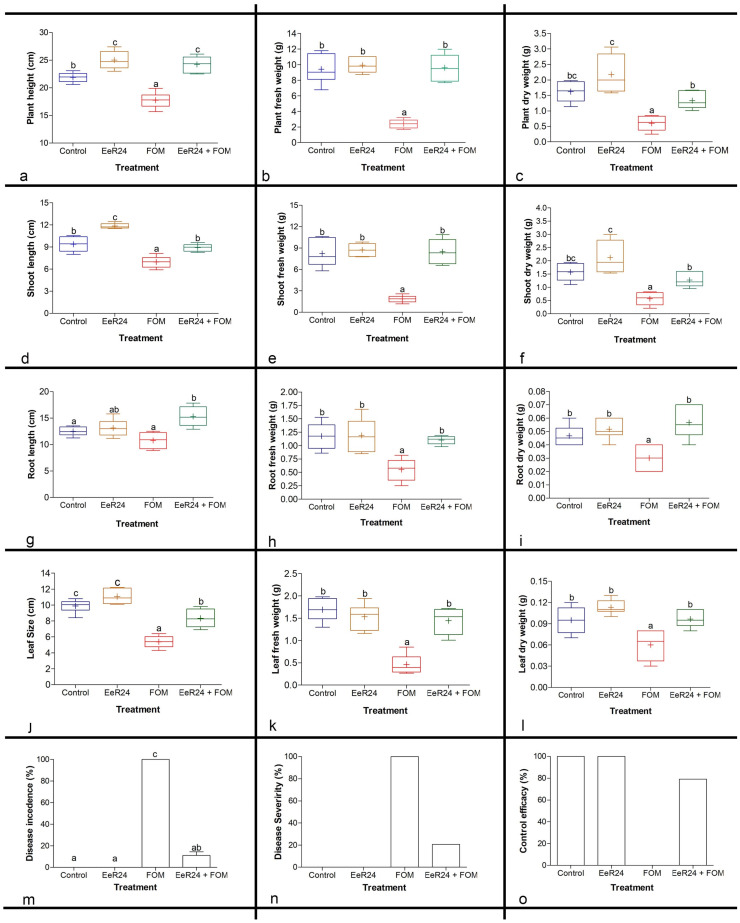
Effect of fungal endophyte *Fusarium clavum* EeR24-colonization on morphological parameters: (**a**) plant height, (**b**) plant fresh weight, (**c**) plant dry weight, (**d**) shoot length, (**e**) shoot fresh weight, (**f**) shoot dry weight, (**g**) root length, (**h**) root fresh weight, (**i**) root dry weight, (**j**) leaf size, (**k**) leaf fresh weight, (**l**) leaf dry weight in melon plants, (**m**) percentage disease incidence (PDI), (**n**) percentage disease severity (PDS), and (**o**) control efficacy of melon plants with various treatments exposed to *Fusarium oxysporum* f. sp. *melonis* (race 1.2) (21 days postinoculation) under greenhouse conditions. Data presented are means ± standard deviation of six replicate plants per treatment. Means with different letters are significant according to Tukey’s post hoc test (*p* < 0.05). A representative experiment of three is presented.

The infection of melon seedlings with Fom negatively impacted the growth and development of melons; however, colonization with endophytic *F. clavum* EeR24 significantly promoted growth compared to Fom-inoculated and non-inoculated plants, respectively (Figure 2). Endophyte colonization improved plant height by 12.4% and 26.7% in melons with or without Fom colonization, respectively (Figure 3a). On the contrary, plant weight (FW and DW) increased by 74.8% and 55.1% compared to the respective non-inoculated plants with Fom infections (Figure 3b,c). Similarly, colonization with *F. clavum* EeR24 also significantly increased the shoot FW and DW by 78.1% and 55.4%, respectively (Figure 3d–f).

Furthermore, *F*. *clavum* EeR24 increased root branching and leaf size in melons under normal conditions; therefore, colonization results were also evaluated in Fom-infected plants. Significant differences were observed in primary root length between colonized and non-colonized plants, either challenged or non-challenged with Fom. Roots of *F. clavum* EeR24-colonized plants were 19.2% longer than those of non-colonized plants, whereas for Fom-challenged plants, root development of *F*. *clavum* EeR24-colonized plants was 12% longer than their non-colonized counterparts (Figure 3g). A significant increase was also observed in the FW and DW of roots, with an increase of 50% and 47.1%, respectively (Figure 3h,i). Additionally, a considerable decrease was observed in the size, weight, and number of leaves in melon plants infected with Fom compared to *F. clavum* EeR24 colonization on size (35%), FW, and DW (37–68%) of leaves infected with Fom compared to the respective controls (Figure 3j–l).

In greenhouse experiments, 100% plant mortality was observed in Fom-inoculated plants, whereas in *F. clavum* EeR24-colonized plants infected with Fom, significantly lower (11.1%) disease incidence was recorded. Increased disease severity was observed in non-inoculated plants infected with Fom (100%), whereas the disease severity was reduced to 16.7% in *F. clavum* EeR24-colonized plants infected with Fom (Figure 3m,n). It was also observed that colonization with endophytic *F. clavum* EeR24 increased the control efficacy against Fom infection of melon seedlings by 83.3% (Figure 3o).

#### 3.5.2. Determination of Membrane Stability Index (MSI), Relative Water (RWC), Malondialdehyde, Proline Content, and Gas Exchange Parameters

**Figure 4:**
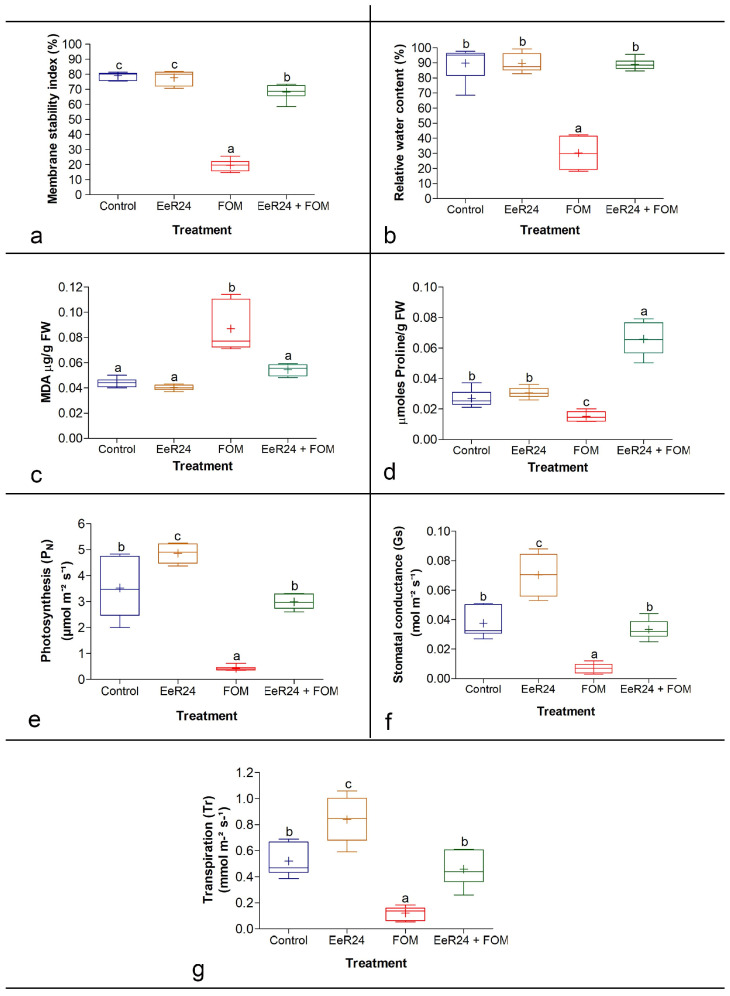
Effect of fungal endophyte *Fusarium clavum* EeR24 colonization on physiological parameters: (**a**) membrane stability index (**b**), relative water content, (**c**) malondialdehyde (MDA) (**d**) proline accumulation (**e**) net photosynthesis, (**f**) stomatal conductance, and (**g**) transpiration in melon plants exposed to *Fusarium oxysporum* f. sp. *melonis* (race 1.2), 21 days postinoculation under greenhouse conditions. Data presented are means ± standard deviation of six replicate plants per treatment. Means with different letters are significant according to Tukey’s post hoc test (*p* < 0.05). A representative experiment of three is presented.

The MSI of melon plants with or without *F. clavum* EeR24 colonization did not differ significantly. However, a significant increase of 71% was observed in the MSI of *F. clavum* EeR24-colonized plants compared to non-colonized plants infected with Fom (Figure 4a). Similarly, no significant differences were observed in the RWC of melon plants under normal conditions, irrespective of their colonization status. However, the RWC of *F. clavum* EeR24-colonized plants infected with Fom increased significantly by 66% compared to the non-colonized plants (Figure 4b).

To further investigate the accumulation of cellular oxidative damage, the aldehyde levels were measured. No significant differences were observed between *F. clavum* EeR24-colonized and non-colonized melon plants. However, a significant increase (1.5-fold) in lipid peroxidation was observed in non-colonized Fom-infected melon plants compared to endophyte-colonized melon plants with Fom infection (Figure 4c).

Similarly, a significant difference in proline accumulation was observed in endophyte-colonized and non-colonized melon plants under normal and inoculated conditions. *F. clavum* EeR24-colonized melon plants exhibited a 1.1-fold increase in proline accumulation compared to non-colonized plants under normal conditions. However, colonization of melon plants with *F*. *clavum* EeR24 mitigated the adverse effect of Fom infection by significantly increasing the proline accumulation 4.3-fold (Figure 4d).

Fom infection reduced the gas exchange properties in melon. Significant differences (*p* < 0.05) in stomatal conductance, transpiration, and photosynthesis were recorded between *F. clavum* EeR24-colonized plants infected with or without Fom. Stomatal conductance (78.5%), transpiration (64.9%), and net photosynthesis rates (85.5%) increased significantly in *F. clavum* EeR24-colonized plants infected with Fom compared to the respective non-colonized plants (Figure 4e–g).

#### 3.5.3. Antioxidant Enzyme Assays

**Figure 5:**
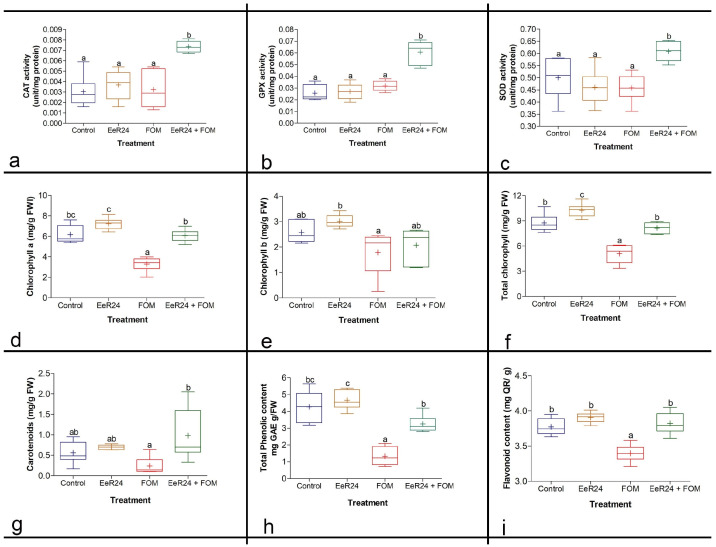
Effect of fungal endophyte *Fusarium clavum* EeR24 colonization on biochemical parameters: (**a**) catalase (CAT), (**b**) guaiacol peroxidase (GPX), (**c**) superoxide dismutase (SOD) activities (**d**) chlorophyll a, (**e**) chlorophyll b, (**f**) total chlorophyll, (**g**) carotenoids (**h**) total phenol, and (**i**) total flavonoid contents in melon plants exposed to *Fusarium oxysporum* f. sp. *melonis* (race 1.2) (21 days postinoculation) under greenhouse conditions. Data presented are means ± standard deviation of six replicate plants per treatment. Means with different letters are significant according to Tukey’s post hoc test (*p* < 0.05). A representative experiment of three is presented.

CAT, GPX, and SOD were recorded in *F. clavum* EeR24-colonized and non-colonized melon plants under normal and inoculated conditions. CAT, GPX, and SOD activities increased significantly in *F. clavum* EeR24-colonized Fom-infected melon plants compared to non-colonized Fom-infected plants. Melon plants colonized with *F. clavum* EeR24 and infected with Fom exhibited a significant increase in CAT (2.3-fold), GPX (1.9-fold), and SOD (1.3-fold) activities compared to non-colonized Fom-infected plants (Figure 5a–c).

#### 3.5.4. Quantification of Total Chlorophyll, Carotenoids, Phenolic, and Flavonoid Content

*F. clavum* EeR24 alleviated the adverse effects of Fom infection in melon plants by increasing chlorophyll (Chl a, Chl b, and total chlorophyll) content under colonized conditions. Colonization of melon plants with *F. clavum* EeR24 increased Chl a by 45.7%, Chl b by 13.8%, and total chlorophyll by 37.6% compared to non-colonized plants with Fom infection (Figure 5d–f). Similarly, total carotenoids, phenol, and flavonoid content of melon plants colonized with *F. clavum* EeR24 and exposed to Fom infections increased significantly compared to non-colonized plants. Endophyte-colonized melon plants exposed to Fom infections exhibited a significant increase in total carotenoid (4.1-fold), total phenol (2.4-fold), and total flavonoid (1.2-fold) content compared to non-colonized control plants (Figure 5g–i).

### 3.6. In Situ Detection of H_2_O_2_ and Superoxide Accumulation, Lipid Peroxidation, and Cell Mortality

**Figure 6:**
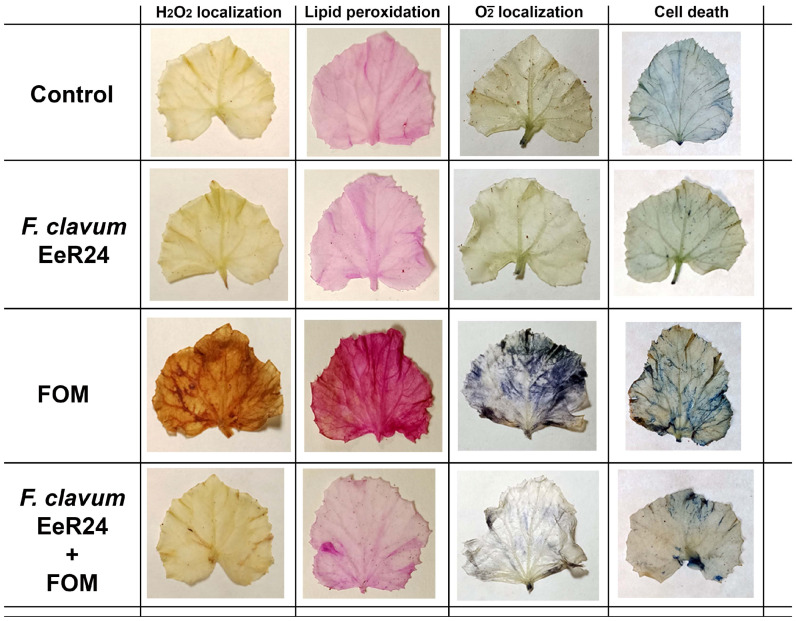
In situ detection of H_2_O_2_, lipid peroxidation, superoxide ions, and hypersensitive cell death in leaves of melon plants colonized by fungal endophyte *Fusarium clavum* EeR24 and then exposed to *Fusarium oxysporum* f. sp. *melonis* (race 1.2).

To determine the in situ accumulation of H_2_O_2_ within the leaf tissues of melon plants, histochemical assays using DAB treatment were performed that enabled the localization of H_2_O_2_ production within the leaf tissues characterized by dark-brown spots. Non-colonized plants exposed to Fom displayed prominent brown spots, in contrast to that of *F. clavum* EeR24-colonized plants under similar conditions (Figure 6). Maximum H_2_O_2_ was recorded in non-colonized plants exposed to Fom while minimum accumulation was observed in *F. clavum* EeR24-colonized plants under normal conditions (Figure 6). Lipid peroxidation was identified by the presence of pinkish spots of MDA on the leaf surface, indicating the formation of lipid peroxides. Elevated levels of lipid peroxidation were characterized by an increased number of pink spots. Among the four treatments, the non-colonized plants exposed to Fom infections showed maximum lipid peroxidation, whereas endophyte-colonized plants exposed to Fom exhibited a reduction in the number of spots (Figure 6). In situ cell death was analyzed according to the number of blue spots formed during hypersensitive reactions. Maximum cell death was observed in non-colonized plants exposed to Fom, characterized by regions of cell mortality on their surfaces. However, colonization with *F. clavum* EeR24 reduced host plant mortality under similar conditions, resulting in decreased blue spots detected by Evans blue staining (Figure 6). Similarly, in situ production of superoxide (O_2_^−^) ions within the leaf tissues was determined using NBT treatment, which enabled the localization of blue formazan deposits produced as a result of reaction with O_2_^−^ ions. Non-colonized plants exposed to Fom resulted in large accumulation of O_2_^−^ ions, whereas *F. clavum* EeR24-colonized plants under similar conditions had substantially reduced O_2_^−^ ion production levels (Figure 6).

### 3.7. Fungal Transformation, GFP and RFP Labeling and Localization of Endophytic F. clavum EeR24 in Melon Seedlings

**Figure 7:**
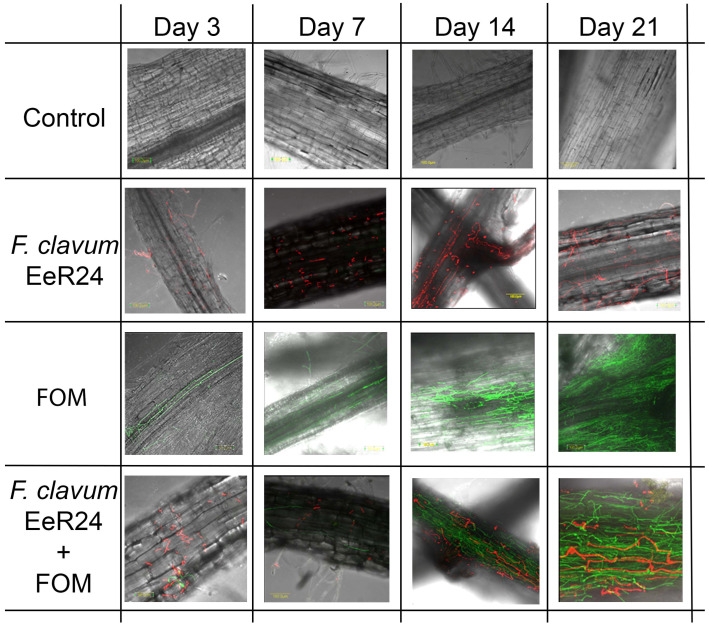
Confocal fluorescent microscopy of roots of melon seedlings colonized by RFP-tagged endophytic *Fusarium clavum* EeR24 after exposure (3, 7, 14 and 21 days) to GFP-tagged *Fusarium oxysporum* f. sp. *melonis*.

Root colonization patterns were evaluated using recombinant Fom and endophytic *F. clavum* EeR24 strains expressing GFP and RFP, respectively. Three days after treating melon seedlings with conidial suspensions, both the GFP- and RFP-expressing fungi had germinated. Their hyphae had elongated by 3 dpi and 7 dpi, and penetration of hyphae was frequently observed in the epidermal cells (Figure 7). Melon seedlings started to exhibit weak green and red signals in various root tissues under normal and inoculated conditions. After 7 days of colonization, increased intensities of GFP and RFP signals were observed during the incubation period. At 14 dpi, Fom formed an extensive hyphal network that covered almost the entire root surface, and strong GFP signals were observed (Figure 7). Furthermore, both Fom and endophytic *F. clavum* EeR24 were observed colonizing the intra- and intercellular tissues of the host plant’s roots, whereas no colonization was observed in stem and leaf tissues (Figure 7). Fom is a fast-growing fungus, exhibiting strong green signals that were recorded in the respective treatments compared to the slower-growing endophytic *F. clavum* EeR24, which exhibited comparatively less red signal. *F. clavum* EeR24 effectively suppressed the pathogenicity of Fom. No fluorescent signals were observed throughout the entire root system of the non-colonized melon plants. This again conformed the endosymbiotic nature of *F. clavum* EeR24 with the host plant and demonstrated its ability to colonize the interior root tissues of melons, as indicated by improving host plant growth and health.

### 3.8. Effect of Culture Filtrate and Organic Residues on Growth of Fom and GC–MS Analysis of Bioactive Residue

**Figure 8:**
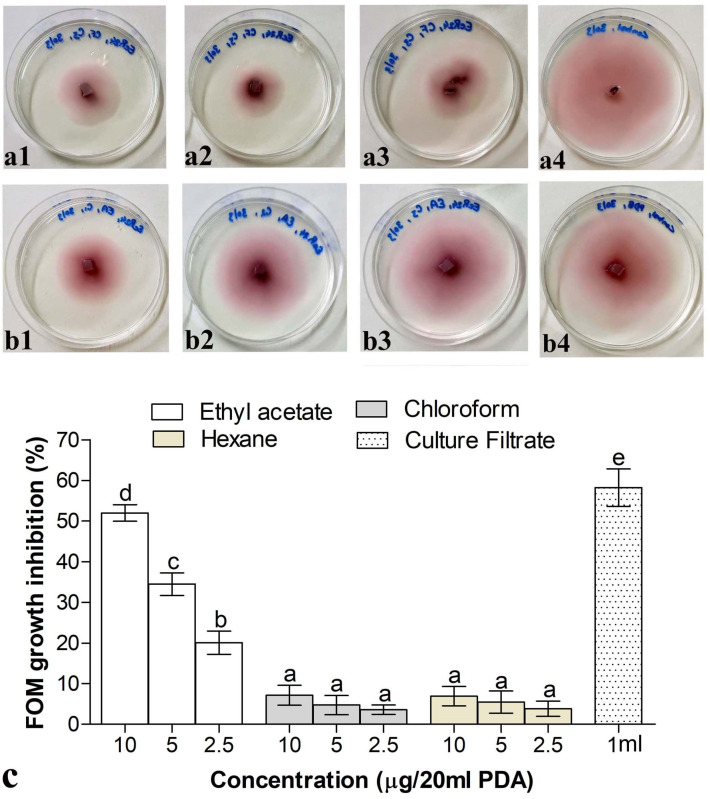
Inhibition of growth of *Fusarium oxysporum* f. sp. *melonis* (race 1.2) by (**a1**–**a3**) culture filtrate and organic residues. (**b1**–**b3**) Ethyl acetate, endophytic *Fusarium clavum* EeR24 in food poison assay with respect to (**a4**,**b4**) control plates without any supplementation. (**c**) Effect of ethyl acetate, chloroform, hexane, and culture filtrate on growth of Fom. Means with different superscript letters are significant by Tukey’s post hoc test (*p* < 0.05).

**Figure 9:**
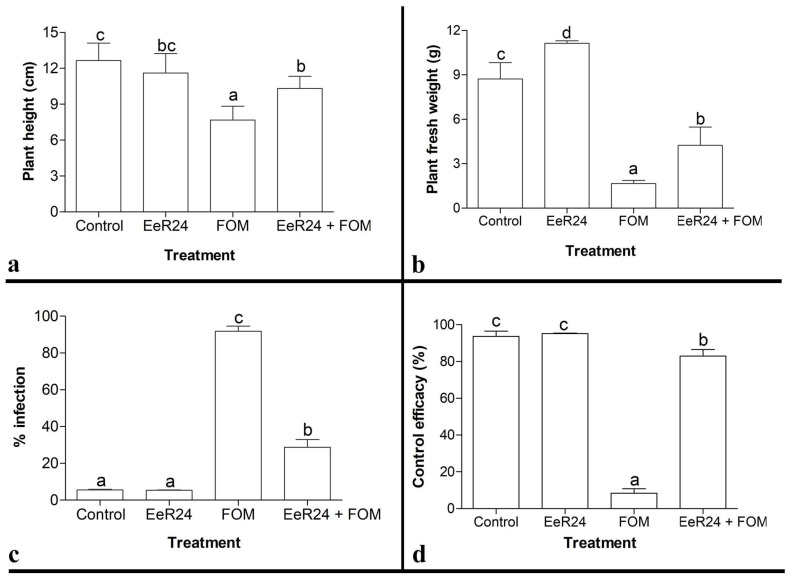
Effect of bioactive organic residue (ethyl acetate) on (**a**) plant height, (**b**) plant weight, (**c**) infection rate, and (**d**) control efficiency in melon plants exposed to *Fusarium oxysporum* f. sp. *melonis* (race 1.2) (21 days postinoculation) under growth chamber assays. Data presented are means ± standard deviation of three replicates. Means with different letters are significant according to Tukey’s post hoc test (*p* < 0.05). A representative experiment of three is presented.

The mycelial growth of Fom was inhibited significantly when incubated along with either culture filtrate or organic residues of *F. clavum* EeR24. The growth of Fom was significantly inhibited by 58% when the medium was supplemented with culture filtrate (Figure 8a,c). Similarly, a dose-dependent inhibition was observed in the growth rate of Fom with an increase in concentration of organic residues. The ethyl acetate residue at 10 µg/20 mL exhibited maximum inhibition (52%) of fungal growth, which decreased gradually (32–20%) as organic residue concentrations decreased. Furthermore, organic residues of chloroform and hexane exhibited weak fungal growth inhibition by 4–7% (Figure 8b,c). In addition, in growth chamber assays, melon plants supplemented with ethyl acetate residue of *F. clavum* EeE24 and exposed to Fom exhibited significantly higher biomass and height compared to non-supplemented plants. After three weeks of exposure to Fom, ethyl acetate residue-supplemented plants increased the height of melon seedlings by 25% and plant biomass by 61% in comparison to non-supplemented plants (Figure 9a,b). Furthermore, the control efficacy of *F. clavum* EeR24 ethyl acetate residue-supplemented plants exposed to Fom was 68% (Figure 9c,d).

Gas chromatography–mass spectroscopy (GC–MS) was used to identify the bioactive compounds in the ethyl acetate residue of *F. clavum* EeR24. Compared to mass spectra in the NIST library, eighteen volatile compounds were obtained based on retention time and molecular weight (Table 3): alpha-hydroxyisobutyric acid, acetate (1), e-11,13-tetradecadien-1-ol (2), dodecanoic acid (3), oxalic acid, allyl hexadecyl ester (4), tetradecanoic acid (5), lauroyl peroxide (6), oxirane, [(dodecyloxy)methyl]- (7), pyrrolo [1,2-a]pyrazine-1,4-dione, hexahydro-3-(2-methylpropyl)- (8), n-hexadecanoic acid (9), 1-ethoxypentan-3-ol (10), 1-propanol, 2-(2-hydroxypropoxy)- (11), carbonic acid, eicosyl prop-1-en-2-yl ester (12), 2-hydroxypentadecyl propanoate (13), 2-propanol, 1-[1-methyl-2-(2-propenyloxy)ethoxy] (14), hexadecane, 1-chloro- (15), 2-piperidinone, n-[4-bromo-n-butyl]- (16), octadecanoic acid, 2-oxo-, methyl ester (17), 4,8-decadienal, and 5,9-dimethyl- (18).

## 4. Discussion

Endophytic fungi have been widely studied for their potential as biocontrol agents against plant pathogenic fungi, as well as their role in promoting plant growth. This approach offers a promising, eco-friendly alternative to chemical fungicides and synthetic fertilizers for improving crop productivity [45,58]. The endophytic fungus *Muscodor albus* has emerged as a promising biofumigant, demonstrating considerable efficacy in the post-harvest disease management of fruit, including apple, grape, citrus, and tomato, by inhibiting major pathogens such as *Botrytis cinerea*, *Penicillium digitatum*, and *Rhizopus* spp. The successful commercialization of this technology by AgraQuest highlights its practical applicability [59]. Similarly, the endophytic bacterium *Bacillus subtilis* QST713 has been developed into the widely used biocontrol product Serenade^TM^, which is extensively applied in fruit crops for the management of diseases such as powdery mildew, brown rot, and late blight [60]. Additionally, the growing interest in endophyte-based formulations is reflected in the development of commercial products such as Candifruit^TM^, Shemer^TM^, and Boni-Protect^TM^, which have shown significant success as biocontrol agents. Collectively, these examples underscore the increasing recognition of endophytes as viable, eco-friendly alternatives to synthetic chemicals for sustainable agricultural practices [61]. Furthermore, several studies have demonstrated the effectiveness of endophytic fungi in controlling wilt diseases [18,42,62,63]. However, research on endophytic fungi isolated from wild cucurbit varieties in hot and arid regions as biocontrol agents against melon pathogens remains limited [6,7]. The present study showed that fungal endophytes isolated from the wild cucurbit *E. elaterium*, which grows natively in the Arava Valley under continuous biotic and abiotic stresses, successfully conferred resistance against Fom wilt in cultured muskmelon. Among the fungal genera isolated in this work, *Alternaria, Cladosporium, Curvularia, Fusarium*, and *Phoma* have also been reported as endophytes in other melon plants [42,64]. This study also revealed a high frequency of *Fusarium* isolates. The elevated occurrence of *Fusarium* species as endophytes in melon suggests a resistance relationship that attenuates fungal virulence, along with a possible balanced antagonism between the plant and fungus, as proposed by [65]. Although the genus *Fusarium* is associated with wilting and other diseases in many important crops, various *Fusarium* spp., when isolated as endophytes, confer protection against plant pathogens such as *Verticillium dahliae, Pythium ultimum*, and *Rhizoctonia solani* [42,66].

In the present study, endophytic fungi were screened for their potential to inhibit the growth of Fom and promote plant growth. The novelty of our study lies in isolating these endophytes from wild squirting melons (*E. elaterium*) growing in the hot, arid conditions of Israel’s Arava Desert. Previous studies have shown that the beneficial effects of endophytes are not limited to their original host and can be transferred to related cultivars by introducing endophytes isolated from wild relatives as bioinoculants [67]. This suggests that endophytes are often defined by their geographic location rather than their functional relationship with the host plant, unlike mycorrhizae, which implies a specific symbiotic interaction [31]. *Fusarium clavum* has previously been isolated as an endophyte from various host tissues worldwide, including roots of potato and wheat [68,69], stems of *Orobanche* spp., and flowers of *Syzygium cordatum* [70,71], where it exhibits diverse bioactivities. Endophytic *Fusarium* species are also capable of coexisting with other beneficial rhizospheric microorganisms, forming a dynamic microbial consortium that may significantly influence plant health. Recent studies indicated that these endophytes interact with bacteria such as *Bacillus, Pseudomonas*, and *Flavobacterium*, which can suppress pathogenic *Fusarium* species through antibiosis, niche competition, and modulation of root exudates [72,73]. Moreover, nonpathogenic *F. oxysporum* strain can function as protective endophytes, enhancing plant resistance against *Verticillium* while maintaining compatibility with other beneficial microbiota [74]. These findings highlight the dual ecological role of *Fusarium* and its potential exploitation in sustainable biocontrol strategies.

Findings from the in vitro screening assays showed that among the 23 endophytes tested, *Fusarium* spp. and *Alternaria* sp. significantly reduced Fom mycelial growth, with inhibition rates of 30–35%, though effects and inhibition types varied by isolate. Three types of inhibition were observed among the tested isolates: Type A (competition for substrate with deadlock upon mycelial contact), Type B (antibiosis characterized by a deadlock at a distance), and Type C (mycoparasitism involving overgrowth without initial deadlock). Among these, endophytic isolates exhibiting Type B interactions (*Fusarium* sp. EeR4 and *Fusarium clavum* EeR24) were selected for further characterization, as antibiosis confers a distinct advantage over the other interaction types. Such isolates produce inhibitory metabolites that suppress pathogen growth without requiring direct contact, making them particularly valuable. Moreover, these isolates can be utilized directly as biocontrol agents, or their culture filtrates or residual metabolites can be exploited for effective disease management. Similar inhibitory effects have been reported for endophytic fungi isolated from melons and other plants, including *Chaetomium, Colletotrichum*, *Epicoccum*, *Fusarium*, and *Trichoderma* spp. [19,28,30,42,64]. Fungal endophytes control plant pathogens through diverse mechanisms. Endophytic *Trichoderma phayaoense* from Siam weed suppressed gummy blight and wilt in muskmelons caused by *Stagonosporopsis cucurbitacearum* and *Fusarium equiseti*, respectively [7]. Similarly, endophytic *Daldinia* cf. *concentrica* from olive trees in Israel inhibited mold growth on organic dried fruits and prevented *Aspergillus niger* infection in peanuts [75]. Recently, endophytic *Penicillium* sp. UM12 from *Urginea maritima* leaves in Israel inhibited *Alternaria alternata*, *Botrytis cinerea, Fusarium oxysporum*, *Sclerotinia sclerotiorum*, and *Rhizoctonia solani* [76]. Endophytic bacteria such as *Bacillus* sp., *B. amyloliquefaciens*, *B. subtilis*, and *Streptomyces* sp. from *C. melo* in Israel also inhibited Fom races 1 and 2 [77]. These reports indicate that endophytes are a valuable reservoir of bioactive compounds with biocontrol properties against plant pathogens [76,78].

Dual-culture assays are commonly used as an initial screening step for biocontrol strains because pot experiments are time-consuming and costly. Strains showing antagonism in vitro are then evaluated in pot experiments with pathogen inoculation to assess disease control efficacy under *in planta* conditions. However, few studies have examined the correlation between in vitro antagonism and *in planta* disease suppression. Evaluations of biocontrol screening strategies have highlighted significant limitations of in vitro methods [42]. In this study, endophytic *Fusarium* sp. EeR4 exhibited antagonism against Fom in screening assays, but showed no activity in the *in planta* assay, indicating no significant correlation between in vitro antagonism and control of muskmelon *Fusarium* wilt. This aligns with findings from [42,45]. The loss of bioactivity in *Fusarium* sp. EeR4 may stem from nutrient composition and microbial growth stage influencing secondary metabolite secretion, as synthetic media are nutrient-richer than soil or natural environments [79]. This likely explains why *Fusarium* sp. EeR4, which demonstrated antibiosis on plates, failed to inhibit Fom *in planta*. In contrast, endophytic *F. clavum* EeR24 conferred resistance against Fom by promoting seedling growth through enhanced biomass and height. This efficacy may be attributed to strain specific traits, such as siderophore production, IAA synthesis, hydrolytic enzymes, and phosphate solubilization, which facilitate root colonization and trigger plant immunity [5].

In the present study, the fungal endophyte *F. clavum* EeR24 conferred resistance against Fom infection in melons by promoting morphological (Figure 3), physiological (Figure 4), and biochemical (Figure 5) responses. Endophytic fungi enhance plant growth under biotic stress by increasing biomass, leaf, shoot and root length, and synthesis of growth-promoting hormones and metabolites [7,45,80,81]. Plant roots are the primary infection site for Fom, and undergo architectural changes that regulate pathogen colonization and enhance tolerance to infection [82,83]. Increased root branching and weight expand the surface area for nutrient and ion uptake, aiding resistance to fungal pathogens [84]. Melon plants colonized with *F. clavum* EeR24 and challenged with Fom showed increased root length and lateral branching (Figure 3g–i). In contrast, Fom infection alone reduced leaf size and weight (Figure 3j–l), likely due to blocked nutrient and water transport from roots to shoots, causing chlorosis and wilting [85]. Colonization with *F. clavum* EeR24 mitigated these effects, preserving leaf size and weight by limiting Fom root colonization. Moreover, *F. clavum* EeR24 significantly reduced *Fusarium* wilt incidence and severity, boosted root and shoot fresh and dry weight, and improved growth in colonized melons. Endophyte-colonized plants exhibited lower disease incidence (11%) (Figure 3m–o) and reduced severity by 16% compared to non-colonized controls, yielding high control efficacy. This resistance can be attributed to host defenses and antifungal metabolites produced by *F. clavum* EeR24. This observation is consistent with previous reports of reduced disease incidence and severity in banana, cucumber, melon, and tomato when colonized with endophytes [7,18,24,34,43,45].

Fom colonization also induces water deficiency in plant tissues, making leaf RWC a key indicator of stress responses and cellular processes. Our findings confirm prior reports that fungal endophyte colonization not only alleviates stress but also enhances water availability from otherwise inaccessible sources [86,87,88]. Fungal infection increases electrolyte leakage by displacing membrane-bound ions, compromising cellular integrity [89]. In this study, *F. clavum* EeR24-colonized melons challenged with Fom showed lower electrolyte leakage, plasma membrane permeability, lipid peroxidation, and malondialdehyde content compared to non-colonized Fom-challenged plants (Figure 4a,c). As an indicator of oxidative damage, elevated MDA reflects higher ROS production and membrane injury [54,90].

Under stress conditions, osmolytes like proline accumulate to maintain osmotic balance and provide energy for growth and survival. *F. clavum* EeR24-colonized Fom-challenged melons exhibited significant proline accumulation (Figure 4d), consistent with reports of endophytes reducing MDA and increasing proline under stress conditions [54,91]. Phenolic compounds also accumulated as an adaptive defense, aligning with prior studies [63,91,92]. Endophyte colonization further maintained photosynthesis, stomatal conductance, and transpiration rates under Fom challenge (Figure 4e–g), likely by regulating stomatal conductivity for resistance. Similar enhancements in photosynthetic activity have been previously reported for endophytes like *Ustilago esculenta* and *Epichloë typhina* [93,94].

Pathogen stress damages membranes and elevates H_2_O_2_ and O_2_^−^ ROS. Endophyte-colonized melons had lower H_2_O_2_ and O_2_^−^ levels than non-colonized Fom-challenged plants, reducing overall ROS (Figure 5a–c). A Similar effect was exhibited by *Piriformospora indica* in enhancing disease resistance via antioxidants in *Anthurium andraeanum* and *Citrus sinensis* [87,95]. The antioxidant enzymes SOD, CAT, and GPX activate to scavenge ROS, where SOD converts O_2_^−^ to H_2_O_2_, which is then converted to water by CAT and GPX [96]. CAT also mitigates photorespiratory H_2_O_2_, supporting photosynthesis [96], while GPX overexpression improves photoprotection [43]. Herein, endophyte colonization increased CAT, GPX, and SOD activities, likely by enabling synergistic ROS scavenging.

Improved chlorophyll content signals endophyte-induced resistance. Fom infection sharply reduced chlorophyll a, b, and total chlorophyll due to fewer leaves or elevated degrading enzymes [97,98]. *F. clavum* EeR24 colonization restored these levels (Figure 5d–f), enhancing P_N_, Gs, and Tr (Figure 4e–g). Previous studies also showed that an increase in antioxidant enzyme activity reduces levels of ROS and H_2_O_2_ in guard cells, leading to opening of the stomata, improved Gs, and enhanced P_N_ and Tr [96,99].

Fom triggers H_2_O_2_ bursts that inhibit host oxidative responses [100]. High H_2_O_2_ in non-colonized plants likely results from pathogen release at infection sites, while endophyte-colonized plants showed lower levels due to elevated CAT, GPX, and SOD production. Lipid peroxidation, a membrane damage marker [101], was highest in non-colonized Fom-challenged seedlings, with histochemical staining confirming elevated superoxide, cell death, and oxidative stress, contrasting sharply with endophyte-colonized plants [54].

*In planta* colonization of roots by *F. clavum* EeR24 conferred significant fitness benefits to melon plants under the tested conditions, observed using RFP-labeled endophytic *F. clavum* EeR24 and GFP-labeled Fom (Figure 7). Similar observations have been reported for an RFP-tagged endophytic *F. oxysporum* F047 isolate, which effectively colonized tomato roots and induced endophyte-mediated resistance against *Fusarium* wilt [102]. Likewise, a GFP-transformed endophytic *Acremonium implicatum* (Acr-1) isolate colonized the epidermal and cortical tissues of tomato roots and exhibited strong biocontrol activity against *Meliodogyne incognita* [103]. In the present study, microscopic analysis further revealed that roots of melons were heavily colonized with Fom compared to endophytic *F. clavum* EeR24; however, the endophytic colonization greatly reduced the pathogenicity of Fom (Figure 7). This was evident by healthier melon saplings when colonized with *F. clavum* EeR24 compared to wilted saplings when not colonized with endophytes.

Fungal endophytes are prolific producers of a wide range of bioactive metabolites. These metabolites serve various functions. In the present study, cell-free culture filtrate of endophytic *F. clavum* EeR24 demonstrated growth inhibition against Fom and suggested that diffusible metabolites could be responsible for the inhibition of Fom. In addition, ethyl acetate residues of *F. clavum* EeR24 also exhibited strong antifungal activity against Fom.

Both the culture filtrate and its ethyl acetate residue exhibited significant inhibitory activity against Fom (Figure 8). Our findings are in accordance with previous studies where culture filtrates and organic residues of endophytic fungi exhibited inhibitory activity against *Pestalotiopsis theae*, *Colletotrichum camelliae*, *Cladosporium cladosporioides*, and *C. sphaerospermum* [76,104,105]. Furthermore, when the culture filtrate of *F. clavum* EeR24 was applied to melon seedlings, it positively influenced the height and weight of plant and replicated the fitness benefit against Fom, as demonstrated by endophytic colonization. Generally, the direct use of fungal endophytes is preferred to check beneficial effects against biotic and abiotic stresses [5,106]. However, fewer studies have been performed in which culture filtrates or their organic residues were applied instead of living organisms to evaluate the effect on fitness benefits (Figure 9). Endophytic fungal isolates such as *F*. *avenaceum, Sarocladium terricola*, and *Trichoderma ongibrachiatum* species have been found to produce metabolites that increase the germination rate in *Lolium multiflorum* and peanuts, along with inhibition of mycotoxigenic fungi like *Aspergillus flavus*, *A. ochraceus*, and *F. oxysporum,* respectively [106,107]. Further, the GC–MS analysis of the ethyl acetate residue suggested production of a mixture of compounds belonging to the chemical classes of acids, alcohols, esters, and oxides. Compounds such as dodecanoic acid, tetradecanoic acid, and n-hexadecanoic acid identified in the present study have been reported to exhibit significant antifungal activity against plant pathogens, including *Botrytis cinerea*, *F. oxysporum* f. sp. *lycopersici, F. oxysporum* f. sp. *niveum*, and *Phytopthora sojae* [108,109,110]. In a previous study, brefeldin A produced by endophytic *Penicillium brefeldianum* HS-1 inhibited the growth of Fom and root-knot *Meloidogyne incognita* nematodes [31]. Similarly, endophytic *Penicillium* sp. UM12 secretes mycophenolic acids that inhibit the growth of phytopathogenic fungi, whereas the volatile organic compounds produced by *Daldinia* cf. *concentrica* inhibited the growth of molds on dried fruits and eliminated *Aspergillus* infection in peanuts [75,76]. These studies suggest that compounds present in the organic residues have potent biological activities and can be further taken into account as an alternative approach for controlling the growth and spread of Fom, thereby reducing reliance on chemical fertilizers and pesticides and supporting the adoption of sustainable agriculture practices.

In summary, our current findings indicate that *F. clavum* EeR24 successfully established a symbiotic relationship with muskmelons under biotic stress. Endophytic *F. clavum* EeR24 significantly improved the growth of melons challenged with Fom by increasing membrane stability, proline and phenol accumulation, stabilizing photosynthetic machinery, and reducing lipid peroxidation and ROS production via enhanced antioxidant activities. To the best of our knowledge, no report exists regarding an endophytic role of *F. clavum* EeR24 isolated from a desert weed plant conferring fitness benefits to muskmelons.

## 5. Conclusions

From the current study, it is evident that Fom infection poses a severe threat to the growth and development of melon plants. However, colonization with the endophytic fungus *F. clavum* EeR24 significantly reduced the detrimental effects of Fom infection on melon seedlings by enhanced biomass production, proline and phenol content, photosynthetic efficiency, and antioxidant activities, which reduced ROS production and hence lipid peroxidation. These effects help melons to resist Fom infection. Further, the culture filtrate or the ethyl acetate residue can also be applied as a biofungicide as well as a biostimulant of growth. The molecular mechanisms employed by *F. clavum* EeR24 to resist Fom infection in melon are under investigation. Moreover, the study suggested that *F. clavum* EeR24 is a promising endophytic isolate for potential application in agricultural applications and as a biocontrol agent against Fom, which severely damages muskmelons plants by causing wilting. In the future, this isolate should be further developed as a biocontrol agent to reduce the spread of *Fusarium* wilt and improve plant immunity in muskmelons.

## Figures and Tables

**Table 1:** Isolation, identification, and screening of fungal endophytes from *Ecballium elaterium* for antagonistic activity against *Fusarium oxysporum* f. sp. *melonis*.

Endophytic Isolate	Fungal Taxa	NCBI Accession Number	% Inhibition	Type of Interaction
EeR1	*Fusarium* sp.	OQ338834	21.74 ± 0.47 ^def^	C
EeR2	*Alternaria* sp. *	OQ338843	14.49 ± 3.32 ^cde^	C
EeR4	*Fusarium* sp.	OQ504479	3.61 ± 1.2 ^abc^	B
EeR5	*Fusarium* sp.	OQ338835	34.74 ± 3.17 ^g^	A
EeR6	*Plectosphaerella* sp.	OQ338836	6.52 ±0.14 ^abc^	A
EeR7	*Fusarium* sp. ^#^	OQ338837	31.88 ± 3.15 ^fg^	C
EeR8	*Fusarium* sp. ^#^	-	30.39 ± 3.69 ^fg^	C
EeR10	*Fusarium* sp. ^#^	-	31.88 ± 4.42 ^fg^	C
EeR12	*Fusarium* sp.	OQ338838	2.88 ± 1.19 ^ab^	C
EeR13	*Aspergillus* sp.	OQ338847	13.77 ± 2.54 ^bcde^	A
EeR15	*Mortierellales* sp.	OQ338848	5.07 ± 1.26 ^abc^	C
EeR16	*Fusarium* sp.	OQ338839	34.04 ± 1.88 ^g^	C
EeR17	*Fusarium* sp.	OQ338840	31.15 ± 4.4 ^fg^	A
EeR18	*Cladosporium* sp.	OQ338849	10.86 ± 2.05 ^abcd^	A
EeR20	*Curvularia* sp.	OQ338844	23.92 ± 0.52 ^efg^	C
EeR22	*Phoma* sp.	OQ338851	22.46 ± 2.51 ^ef^	C
EeR24	*Fusarium* sp.	ON908966	31.82 ± 5.62 ^fg^	B
EeR25	*Alternaria* sp. *	-	29.61 ± 7.01 ^fg^	C
EeR26	*Cladosporium* sp.	OQ338850	2.16 ± 2.17 ^a^	A
EeR27	*Aspergillus* sp.	OQ504480	22.49 ± 3.48 ^ef^	A
EeR28	*Alternaria* sp. *	-	34.75 ± 3.17 ^g^	C
EeR29	*Alternaria* sp.	OQ338845	7.19 ± 4.83 ^abc^	A
EeR30	*Aspergillus* sp.	OQ338846	9.36 ± 4.36 ^abc^	A

Note: Data presented as means ± standard deviation of three replicates. Means with different letters are significant according to Tukey’s post hoc test (*p* < 0.05). The test was repeated twice and a representative experiment of two is presented. # and * isolates were identical as per Ap-PCR. Type A: competition for substrate; Type B: antibiosis; Type C: mycoparasitism. A = deadlock with mycelial contact, B = deadlock at a distance, C = overgrowth without initial deadlock.

**Table 2:** Effect of endophytic fungal colonization on melon seedlings exposed to *Fusarium oxysporum* f. sp. *melonis* (race 1.2) under growth chamber conditions.

Treatment	Plant Fresh Weight (g)	Plant Height (cm)	Infection (%)	Control Efficacy (%)
Control (reference plant)	20.66 ± 1.53 ^bc^	13.32 ± 1.88 ^d^	-	-
*Fusarium* sp. EeR4	19.33 ± 1.53 ^bc^	12.11 ± 1.31 ^cd^	10.39 ± 0.8 ^a^	89.61 ± 0.8 ^c^
*Fusarium clavum* EeR24	21.33 ± 2.08 ^c^	14.06 ± 3.36 ^d^	6.17 ± 2.22 ^a^	93.83 ± 2.22 ^c^
*Fusarium oxysporum* f. sp. *melonis* (Fom)	1.67 ± 0.58 ^a^	7.28 ± 1.86 a	91.8 ± 3.2 ^c^	8.2 ± 3.2 ^a^
*Fusarium* sp. EeR4 + Fom	2 ± 1 ^a^	8.28 ± 0.5 a^b^	89.88 ± 4.38 ^c^	10.12 ± 4.38 ^a^
*Fusarium clavum* EeR24 + Fom	17 ± 2 ^b^	10.74 ± 0.95 ^bc^	20.39 ± 2.73 ^b^	79.61 ± 2.73 ^b^

Data represent means ± standard deviation of three replicates after 21 days postinoculation. Means with different letters are significant according to Tukey’s post hoc test (*p* < 0.05). A representative experiment of three is presented. -: No activity.

**Table 3:** GC–MS spectrum of the bioactive ethyl acetate residue.

Compound	Formula	Score (%)	Mass
Alpha-hydroxyisobutyric acid, acetate	C_6_H_10_O_4_	66.93	146.1
E-11,13-tetradecadien-1-ol	C_14_H_26_O	84.09	210.2
Dodecanoic acid	C_12_H_24_O_2_	77.96	200.2
Oxalic acid, allyl hexadecyl ester	C_21_H_38_O_4_	80.11	354.3
Tetradecanoic acid	C_14_H_28_O_2_	74.2	228.2
Lauroyl peroxide	C_24_H_46_O_4_	65.57	398.3
Oxirane, [(dodecyloxy)methyl]-	C_15_H_30_O_2_	70.78	242.2
Pyrrolo[1,2-a]pyrazine-1,4-dione, hexahydro-3-(2-methylpropyl)-	C_11_H_18_N_2_O_2_	72.47	210.1
n-Hexadecanoic acid	C_16_H_32_O_2_	78.04	256.2
1-Ethoxypentan-3-ol	C_7_H_16_O_2_	69.38	132.1
1-Propanol, 2-(2-hydroxypropoxy)-	C_6_H_14_O_3_	69.52	134.1
Carbonic acid, eicosyl prop-1-en-2-yl ester	C_24_H_46_O_3_	76.73	382.3
2-Hydroxypentadecyl propanoate	C_18_H_36_O_3_	78.02	300.3
2-Propanol, 1-[1-methyl-2-(2-propenyloxy)ethoxy]	C_9_H_18_O_3_	71.47	174.1
Hexadecane, 1-chloro-	C_16_H_33_Cl	65.32	260.2
2-Piperidinone, N-[4-bromo-n-butyl]-	C_9_H_16_BrNO	64.96	233
Octadecanoic acid, 2-oxo-, methyl ester	C_19_H_36_O_3_	62.38	312.3
4,8-Decadienal, 5,9-dimethyl-	C_12_H_20_O	68.65	180.2

Note: The GC–MS technique can primarily detect low-molecular-weight and volatile compounds. *Fusarium* spp. are known to produce an array of secondary metabolites, including antibiotics and toxins, which are nonvolatile and may not be detected using this technique. Therefore, further comprehensive analyses involving complementary methods (LC-MS, HPLC, etc.) are warranted for complete characterization and better understanding of the antifungal activity of these compounds.

## Data Availability

Data will be made available upon request.

## Supplementary Materials

The following supporting information can be downloaded at: https://www.mdpi.com/article/10.3390/microorganisms14040871/s1, Figure S1: Band pattern of Arbitrarily primed PCR (ap-PCR) amplified genomic DNA of *Endophytic isolates* of *Ecballium elaterium* using three primers (CAG)_5_, (GACA)_4_ and (GACA)_3_. M: DNA molecular marker, C: Control.
