# Supplementary material for: Habitat-Adapted Endophytic Fusarium clavum EeR24 from the Arava Desert Induces Resistance Against Fusarium Wilt of Muskmelons
Source: Microorganisms. 2026 Apr 12;14(4):871. doi: 10.3390/microorganisms14040871 (PMC13119043; doi:10.3390/microorganisms14040871)
Supplement: Supplementary file 1 [file microorganisms-14-00871-s001.zip › microorganisms-4161815-supplementary.pdf]

Figure S1: Band pattern of Arbitrarily primed PCR (ap-PCR) amplified genomic DNA of endophytic isolates of *Ecballium elaterium* using three primers $(CAG)_5$, $(GACA)_4$ and $(GACA)_3$. M: DNA molecular marker, C: Control.

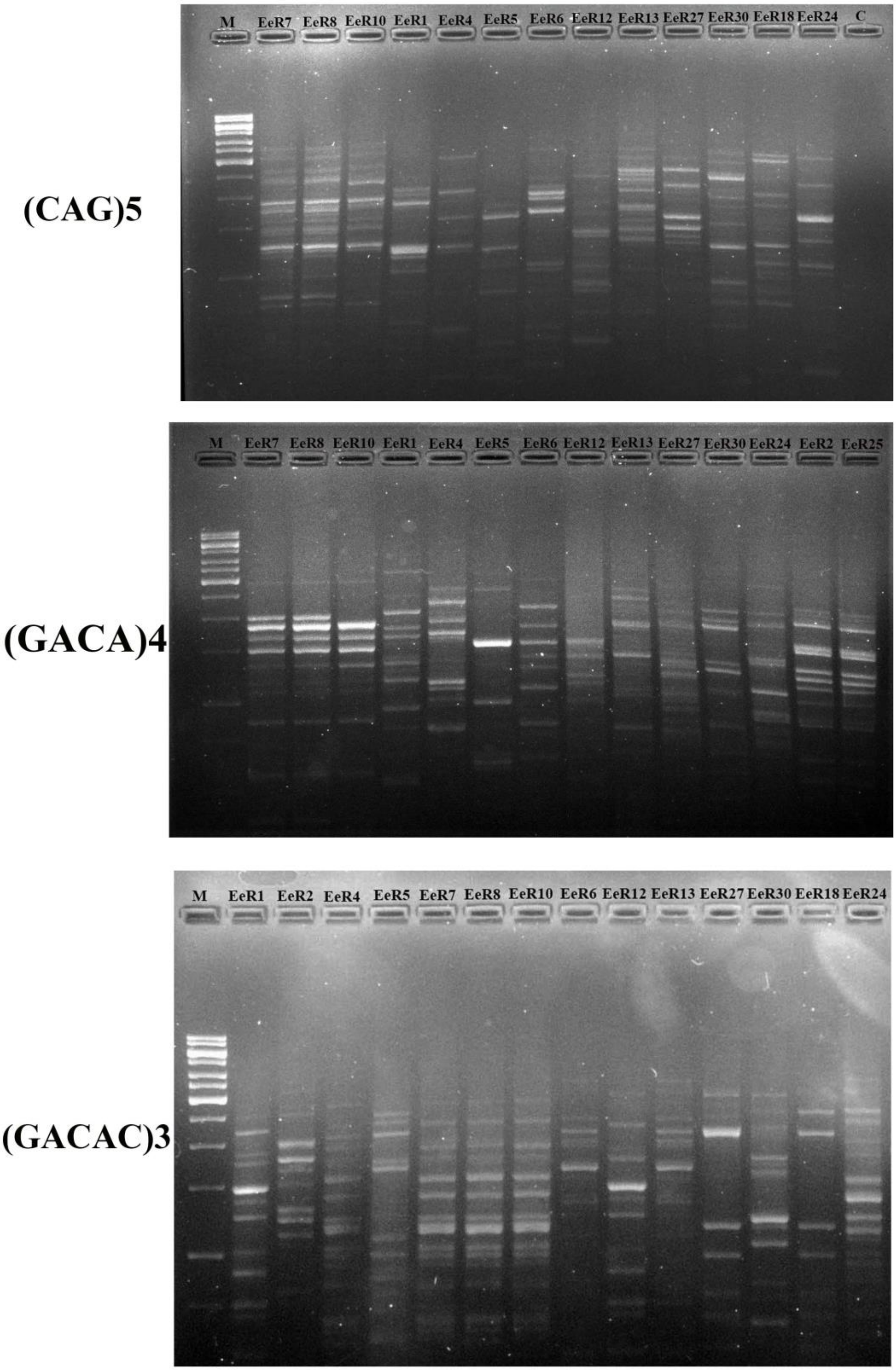

M: Molecular marker (1Kb); C: Control
